# Women’s Leadership Development at the Yale School of Medicine: Preliminary Evaluation of an Innovative Program

**DOI:** 10.1089/whr.2024.0188

**Published:** 2025-03-18

**Authors:** Daryn H. David, Ishita S. Arora, Azza Hussein, Jessica Gois Santana, Cindy A. Crusto, Darin Latimore

**Affiliations:** ^1^ Director for Leadership Development & Coaching Initiatives, Offices of Academic & Professional Development (OAPD) and Diversity, Equity, & Inclusion (ODEI), Yale School of Medicine, Professional Certified Coach Through the International Coaching Federation, Yale Child Study Center, New Haven, Connecticut, USA.; ^2^ Equity Research and Innovation Center (ERIC), Office of Health Equity Research (OHER), Department of Internal Medicine, Yale School of Medicine, New Haven, Connecticut, USA.; ^3^ American Psychiatric Association, Washington, District of Columbia, USA.; ^4^ Department of Biomedical Engineering, School of Engineering & Applied Science, Yale University, New Haven, Connecticut, USA.; ^5^ Department of Psychiatry and the Behavioral Sciences, Senior Associate Dean for Program Development, Effectiveness, and Evaluation, Office of Faculty Affairs, Advancement, and Inclusion, Keck School of Medicine of the University of Southern California, Los Angeles, California, USA.; ^6^ Section of General Internal Medicine, Deputy Dean for Diversity and Inclusion and Chief Diversity Officer, Yale School of Medicine, New Haven, Connecticut, USA.

**Keywords:** academic medicine, coach approach, leadership development, women faculty

## Abstract

**Background:**

Despite strong data indicating women leaders’ proven efficacy as catalysts for organizational change, there is significant attrition for women across the advancement, promotion, and leadership pathways within academic medicine. To help early-career women faculty build a network of support, enhance leadership capacity and agility, and gain the skills necessary for career advancement and fulfillment, we created the Women’s Leadership Development Program (WLDP) at our medical school in 2020.

**Methods:**

From 2023 to 2024, we collected retrospective survey data from all interested prior participants to gauge the impact of the WLDP on faculty members’ confidence, sense of belonging at Yale School of Medicine, and acquisition of leadership skills.

**Results:**

Findings indicate the WLDP’s positive impact on women faculty members’ sense of leadership efficacy and skills, with participants reporting enhanced appreciation of their leadership potential, knowing how to lead with their strengths, improved leadership vision, and increased confidence in making an impact in academic medicine going forward.

**Conclusion:**

The importance of leadership development programming for the professional development of early-career women faculty in academic medicine is explored. The article concludes with implications of these findings for our ongoing programming and leadership development initiatives for women in academic medicine more broadly.

## Introduction

Although women constitute the majority of matriculants to medical school (55% as of 2023–2024) and currently make up 37% of physicians, they are represented in ever-declining numbers as they progress along the ranks of academic medicine.^1–4^ Women also are much less likely to obtain leadership positions, holding 18% of department chair and full deanship positions, respectively,^5^ and tend to earn considerably less money than their male counterparts in these roles, especially at private medical schools.^6^ Taken together, these figures continue the trend toward career stagnation found from 1979 to 2013, during which women faculty remained consistently less likely than their male counterparts to be promoted from assistant professor or to take on departmental chair positions.^7^

These disparities in advancement, promotion, and leadership exist despite data indicating women leaders’ efficiency as catalysts of organizational culture change.^8^ Women’s leadership on clinical teams has been found to enhance patient outcomes postoperatively,^9–10^ and women’s involvement on research teams has been correlated with higher quality scientific output.^11^ Female leaders can serve as powerful role models within academic medicine, helping to retain other women faculty and setting an example of confident leadership.^12–13^ Other benefits include advancements in health equity research, teaching innovation, improvements to financial performance, and greater access to treatment among underserved communities.^14^

The barriers to women’s advancement in academic medicine are multifaceted and rooted in long-standing internal and external challenges. Women faculty have lower starting salaries across subspecialities; inequitable access to resources and supports; and report dissatisfaction with their rate of advancement while also feeling too overloaded with work to pursue avenues for leadership and promotion.^15–18^ Like other women professionals, they wrestle with internalized gender norms, including the pressure to take on a greater proportion of domestic and childcare responsibilities.^18–19^ These and other barriers help foster higher rates of burnout among female physicians and exacerbate the “leaky pipeline,” or loss of women faculty over the course of the academic career lifecycle, especially as they just begin to make their professional mark.^14,20^

Workplace gender biases, whether implicit or explicit, also presents a significant obstacle for the advancement. Health care professionals, including those in academic medicine, often exhibit unconscious biases against women providers,^21^ with women leaders considered less effective than men and forced to navigate self-doubt about their efficacy.^22^ Studies have likewise revealed gender biases in letters of recommendation written for female versus male faculty members;^23^ in pay for commensurate clinical work^24^; during clinical encounters, with female attending physicians going unacknowledged more often than their male counterparts^25^; and during presentations at some annual society meetings, during which male introducers refer less frequently to women presenters by their titles.^26^ Further, many institutions view programs targeting the recruitment, promotion, and retention of women in academic medicine as nonessential,^27^ an approach we believe can only serve to exacerbate gender-based disparities and the detriment to research and clinical practice that nondiverse teams present.^10,28^ Interestingly, highlighting institutional career flexibility policies may also backfire, with more women voicing concerns that colleagues will negatively evaluate their career commitment if they take caretaking leave.^29^

Thankfully, the situation is not entirely bleak. In recent years, various industries including academic medical centers have come to embrace the role that women’s leadership development initiatives can play in promoting career advancement and success.^30^ Although such programs alone cannot resolve systemic and structural issues of gender inequity,^30^ a review of the literature reveals the beneficial impact they can have on career promotion, women’s sense of satisfaction and career efficacy, and the development of their networks within academic medicine. Below is a review highlighting the promise of this approach.

### Review of existing programs

Targeted professional and leadership development programs have demonstrated positive outcomes for women faculty in academic medicine.^31^ Assuming many different forms, these programs provide support by fostering community, teaching essential skills, and normalizing the challenges that women faculty face.

One key benefit concerns reductions in isolation and enhancements to peer support and networking.^32^ For example, participants in a pilot program at the Baylor Scott and White Medical Center strongly endorsed the benefits of camaraderie and networking that the program afforded.^33^ Senior women faculty who have completed the esteemed Hedwig van Ameringen Executive Leadership in Academic Medicine (ELAM) program, which aims to comprehensively train associate and full professors to assume leadership positions, likewise report networking, interpersonal support, and confidence in leadership skills as key gains.^34–35^

Such programs may also foster the acquisition of leadership skills. After completing the skills-focused Leadership Program for Women Faculty at Johns Hopkins University School of Medicine, women faculty reported improved capacity to handle crucial conversations, deal with difficult behavior, negotiate, and influence others; they also appreciated the chance to network with other women.^36^ A mid-career development program for women faculty at the University of Rochester Medical Center focused on enhancing effective communication strategies and awareness of promotion and institutional infrastructure; postprogram, most participants reporting having reached their self-identified goals concerning leadership, networking, career development, and negotiation, with many graduates eventually assuming leadership roles within their institution.^37^

Particularly heartening are the data speaking to the positive impact of such interventions for early-career women faculty. Pre/post evaluation of the recently adapted Johns Hopkins program—now termed the Early Career Women’s Leadership Development Program—reveals enhanced confidence in leadership skills, vision, and ability to foster personal/professional values alignment among participants.^38^ Further, a review of three US career development programs designed for women faculty indicates that participating women assistant professors had higher rates of retention in academic medicine, especially during the first decade or so after appointment, than did rank-compatible male and nonparticipating women faculty.^39^ Given the changes occurring within academic medicine, including novel interdependencies between the academic and health care spheres and the evolving leadership opportunities these may create, we agree with Chaudron et al. that teaching leadership skills to early-career women could prove more critical than ever.^40^

To this point, women's leadership development programs also evidence a direct positive impact on promotion and advancement. The Faculty Scholars program at the University of Missouri Kansas City School of Medicine guides participants to identify strengths and development opportunities and cultivate mentorship and sponsorship relationships. Initial participants reported career promotion and the assumption of new leadership roles.^14^ Self-reports of advancement and promotion were also found among participants in the Medical University of South Carolina’s career development program, which provided women faculty with information, skills, and resources needed for promotion, tenure, and leadership development.^41^ Further, a disproportionately large assumption of school and department-level leadership roles was evidenced by women participants across 12 years of the UCSF-Coro Faculty Leadership Collaborative, which was open to male and female faculty across the schools of Dentistry, Nursing, Medicine, and Pharmacy.^42^

Finally, programs that focus on systemic change also have an important role to play.^42^ In addition to its emphasis on networking and skills-building, the ELAM program supports participants in seeding empirically validated interventions that promote gender equity within their institutions.^35^ Calls to action to bring about sustained cultural change, enhanced equity, and support for women’s careers in academic medicine also abound.^43^

### Pillars of the Yale School of Medicine Women’s Leadership Development Program

Inspired by the promising interventions described above, we will now introduce the Yale School of Medicine Women’s Leadership Development Program (YSM WLDP). The focus of the WLDP is on helping early-career women faculty build a support network with one another, enhance their leadership capacity, and gain skills and comfort navigating career challenges. Additional objectives include growing the leadership capacity and vision of early-career women faculty at YSM and equipping early-career women faculty with tools to navigate the leadership structure at YSM. For this program, early career refers to women faculty who have been in their current positions for 6 or fewer years and are assistant professors and/or early career on the research track.

A confluence of factors led to the birth of the YSM WLDP in the fall of 2020. First was the strong top-down interest by one of our departments in creating a program that would help women faculty gain the necessary skills and navigate gender stereotypes as they progressed through their careers. Running alongside this was a commitment by YSM leadership to support the leadership development of women faculty. Drawing momentum from these streams of interest, the nascent YSM WLDP was developed by the first author in collaboration with other YSM colleagues. The program has been deployed without interruption once every semester, with one cohort per semester, for a total of eight semesters/cohorts through the spring of 2024.

The following conceptual pillars guided the development of the program. They are drawn from the leadership development literature and also stem from our intention of helping early-career women faculty progressively shift from an “apprenticeship mindset”^44^ of working in accordance with the demands of institutional pressure to one of greater self-determination, professional presence, generative interdependence, and authentic voice.

*Pillar #1: Anchoring oneself.* The YSM WLDP rests on the premise that once an individual knows her strengths and core motivators for doing her work, she can engage with her responsibilities from a place of greater intrinsic motivation, confidence, and choice. This premise resonates with the notion of the “inner compass”—composed of values, self-awareness, strengths, motivations, and interpersonal balance—that help one to align with her “True North,” or deepest reason for doing and being.^45^ The WLDP utilizes self-assessment instruments including the CliftonStrengths^46^ and Everything DiSC Workplace,^47^ to help participants identify their strengths, style of working, and the deeper “why” or sense of calling that they bring to their work. The last meeting of the program comes full circle by including an Inner Mentor guided meditation, designed to connect participants to deeper wells of inner wisdom and power within themselves.^48^

*Pillar #2: Cultivating skills and relationships.* The leadership skills necessary for managing interpersonal relationships intelligently and effectively—skills grounded in an emotional intelligence that is foundational to effective leadership—are taught in the middle of the program.^49^ These include deep listening, handling difficult conversations, and taking a developmental approach toward delegation and managing up. Techniques for managing time and setting priorities are also introduced.

Further, participants are encouraged to take a peer coaching approach of thought-provoking questioning-rather than direct advice-giving—as they support each other in practicing these new skills.^50^ Such an approach entails the “coach” engaging in deep listening and open-ended questioning intended to help the coachee to home in on a current challenge, articulate where she would like to be with it in the future, and identify steps required to achieve this desired end state.^44^ The integration of coaching into women's leadership development programs is not new; what we believe is newly emerging in our program and others is the concerted teaching of coaching mindsets and skills to participants, so that they may become equipped to help themselves and one another using a proactive and empowering problem-solving approach.^38^

*Pillar #3: Embracing connection and generativity.* In the world of academic medicine, individualistic accomplishment remains the dominant metric of success. In the WLDP, we both acknowledge that ethos and work to soften it by deliberately stressing the importance of generativity and connection with others. Specifically, drawing on psychologist Erik Erikson’s work, we define generativity as the capacity to work in the service of aims beyond oneself, with an openness and capacity to uplift and develop others.^51^ We define connection as a willingness to hold one’s own insecurities and wounds while approaching oneself and others with compassion, curiosity, and grace.^52–53^

Toward the end of the course, participants are supported in thinking more broadly about how to bring their own values, professional desires, and presence more fully into the workplace, during moments of concordance and discordance between personal perspectives and institutional exigencies. They are likewise encouraged to identify patterns of speech and mind that may diminish their standing, while incorporating a spirit of serving others to more fully and comfortably claim their own accomplishments and contributions.^48^ We reemphasize the centrality of peer support by encouraging participants to maintain supportive ties with one another following the program’s completion.

## Methods and Materials

The YSM WLDP unfolds across five, 2-hour group sessions that provide a mix of didactic and experiential instruction. Course content is designed to help faculty acquire foundational communication and organizational skills, establish career goals, become familiar with peer coaching, and articulate leadership vision and purpose. Participants are encouraged to build enduring, supportive relationships with one another and identify how to further grow their professional networks.

Readings and other tasks are assigned between sessions, which occur virtually approximately once every two to three weeks. Sessions are run through Zoom and the NovoEd^54^ learning management system, a collaborative, instructional interface that enables real-time exchange of materials, comments, and collection of survey data. The WLDP culminates in each participant’s creation of an individualized professional development plan articulating current, actionable leadership goals.

Stemming from the premise that gaining fundamental leadership skills at the beginning of one’s career can yield fruit for years to come, recruitment has focused on early career faculty. The first group of participants was nominated by three departments known for their support of women faculty. The group was comprised of 11 women faculty total, split into two cohorts/semesters. Each cohort received four, 2-hour sessions designed to teach foundational skills in communication, delegation, and mentorship; setting priorities and time management; and articulating leadership vision and purpose. Each participant also received 2 hourlong, one-on-one coaching sessions with the first author, prior to and following the course meetings.

Following the positive reception of the program by members of the first two cohorts, we decided to open enrollment to women faculty across departments at our medical school. The criteria for eligibility included early-career status and the intention to attend all sessions of the program. Upon expansion, it became apparent that coaching resources constraints would make it impossible to maintain the 1:1 coaching sessions and also scale the program. Thus, beginning with cohort 3 in fall 2021, the structure of the program shifted to five, 2-hour sessions without individual coaching sessions. In addition to prior topics covered, the curriculum was expanded to include an introduction to peer coaching skills, coupled with ample opportunities for participants to practice these skills and build enduring, supportive relationships with one another. Recruitment efforts involved an open-call to all early-career women faculty across YSM.

The only deviation from this new structure involved cohort 8. When the first author had to cancel one session due to an unavoidable conflict, she gave a 30-minute coaching session focused on relevant course topics to any participant (*n* = 4) who could not attend the rescheduled session.

### Detailed program content

The WLDP course material is divided into the following buckets:

- Leading with assets and strengths.
- Communicating to lead others.
- Getting it all done, with minimal angst.
- Nurturing professional relationships.
- Moving forward with wisdom and presence.

As indicated in Table 1, this content is delivered sequentially, across the five course sessions.

**Table 1.** Breakdown of Women’s Leadership Development Program Sessions

Session	Content/Skills	Preassessments/In-session activities
1. Leading with Assets and Strengths	Establishing group norms/confidentiality Identifying and leading with our strengths Identifying purpose and sense of “Why”	Self-assessment of strengths (CliftonStrengths)^46^ and partner activity exploring strengths
2. Communicating to Lead Others	Essential listening skills Navigating crucial conversations Fundamentals of peer coaching	*Coach Approach conversation #1,* focused on utilizing listening and inquiry skills to help partner home in on areas of communication they would like to develop
3. Getting it All Done, with Minimal Angst	Identifying preferred work and communication styles Setting priorities Time management Coaching refresher	Self-assessment of workplace style (Everything DiSC Workplace).^47^; preparation of log detailing time spent on various work activities *Coach Approach conversation #2,* focused on supporting partner to align priorities with time spent on different work activities
4. Nurturing Professional Relationships	Stakeholder mapping Delegation Mentorship Building networks Conscious accountability	Self-assessment and peer conversation focused on optimizing relationships at work
5. Moving Forward: Wisdom & Presence	Tapping inner mentor Articulating values (personal and institutional) Harnessing voice and presence	Inner mentor meditation.^48^ and group exploration of speaking styles and ownership over accomplishments
Postprogram	Completion of individual development plan	

### Approach to evaluation

Evaluation refers to the systematic collection of information about the activities, characteristics, and outcomes of programs to gauge impact, improve effectiveness, and inform decisions about future program development for continuous quality improvement.^55–56^ To collect evidence regarding how the WLDP might affect women’s leadership at YSM, we developed the following retrospective evaluation.^57^ The study was approved by Yale’s Human Investigation Committee (Institutional Review Board Protocol 2000034795) and conducted in 2023–24.

**FIG. 1.**
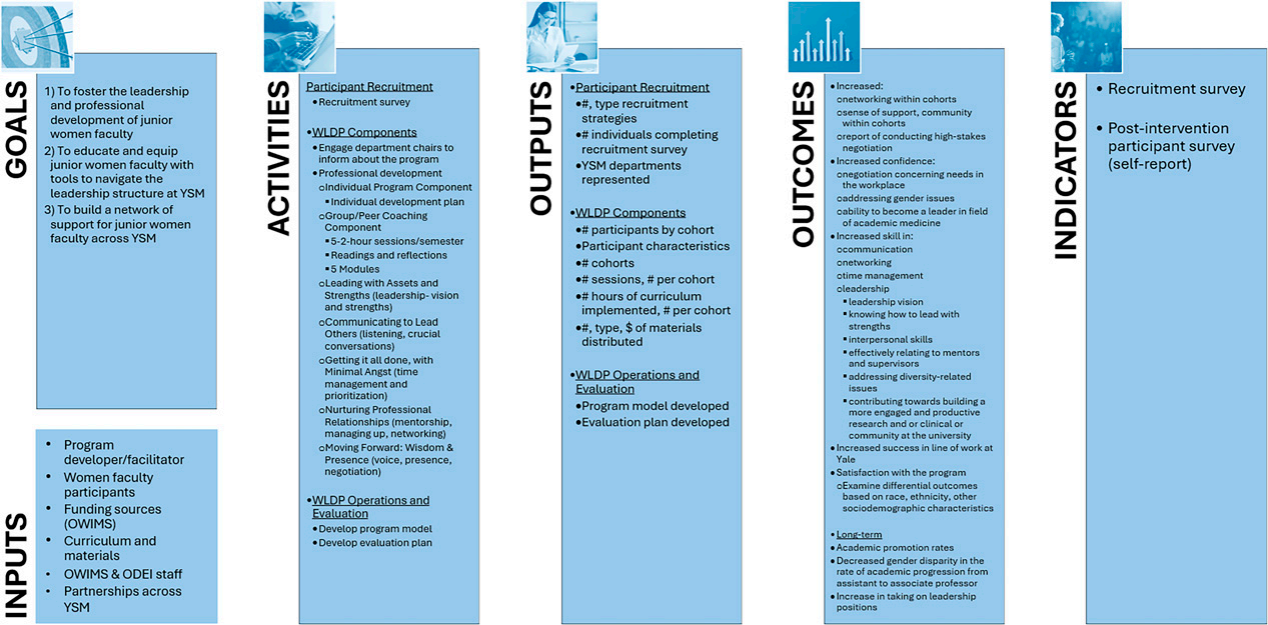
Logic Model for Retrospective Evaluation of YSM WLDP, Yale School of Medicine Women’s Leadership Development Program.

First, we created a logic model in collaboration with key stakeholders. A logic model is a dynamic representation of a program’s theory designed to aid determination of how all program components (*i.e.,* resources, activities/interventions, deliverables, and outcomes) work together to affect the desired changes.^58^ As indicated in our logic model (Fig. 1) the three key goals of the WLDP are to (1) foster the leadership and professional development of early career women faculty; (2) educate and equip these faculty with tools to navigate the YSM leadership structure; and (3) help these faculty build an internal network of support.

An email originating from the study’s Principal Investigator and then Director of Office for Women in Medicine and Science at YSM (*i.e.,* the second-to-last author on this paper) invited all past members of the YSM WLDP cohorts (from fall 2020 to spring 2024) to fill out the evaluation. Data were collected through anonymous links to an online survey on the Qualtrics Survey Tool platform.^59^ Before beginning the survey, participants were given complete information about the study purpose and procedures through an online informed consent form. No personal identifying information (such as name, birthdate) was collected; likewise, any collected information that posed risk of identification was later redacted, and all responses were held in confidence.

The survey contained questions focused on participant demographics and their experience with the WLDP. Please see Table 2 for a listing of the most salient demographic variables, and Table 3 for a breakdown of participation in the study by academic department and cohort number. Questions regarding the program included ten Likert-scale items gauging participants’ satisfaction with the WLDP, their experience with the program instructor, and their leadership pursuits after program completion. Also included were several open-ended qualitative items focused on participants’ perceived leadership capacity and their overall impressions. Please see Table 4 for a listing of all survey items.

**Table 2.** Demographic Results (*n* = 26)

Variable	*n*
Age	
25–34 years	2
35–44 years	21
45–54 years	3
LGBTQIA+	
No	24
Yes	1
Prefer not to say	1
Race	
Asian or Asian American	6
Black or African American	3
White	14
Other/Multiracial	3
Ethnicity	
Latino/a/x or Hispanic	2
Non-Hispanic or Latino/a/x	24
Citizenship	
U.S. citizen	21
Non-U.S. citizen	5
Faculty	
Full-time	25
Part-time	1
Rank	
Assistant professor	18
Associate professor	3^a^
Professor	1
Research scientist	2
Associate Research scientist	1
Other	1
Been through appointments and promotion process	
Yes	12
No	14
Prior experience with any leadership course	
Yes	12
No	14
Prior experience with group or individual leadership development/career coaching	
Yes	9
No	17

^a^ Two of these associate professors were assistant professors at the time of participation in the WLDP. They were promoted between their participation and the collection of study data. The third faculty member answered “1 year” in the associate professor position upon completing the survey 8 months after program participation. Depending on whether they rounded their response up, this person therefore may have received a promotion a month prior to, during participation, or shortly after joining the YSM WLDP.

YSM WLDP, Yale School of Medicine Women’s Leadership Development Program.

**Table 3.** Participation by Department and Women’s Leadership Development Program Cohort (*n* = 26)

Variable	*n*	Percentage of sample
Department		
Genetics	1	3.8
Internal medicine	2	7.7
Neurology	1	3.8
Gynecology and reproductive sciences	2	7.7
Pediatrics	2	7.7
Psychiatry	4	15.4
Surgery	1	3.8
Therapeutic radiology	1	3,8
Urology	1	3.8
Public health	3	11.5
Other	1	3.8
No Answer/Unknown	7	26.9
Cohort		
# 1	2	7.7
# 2	0	0.0
# 3	3	11.5
# 4	3	11.5
# 5	6	23.1
# 6	4	15.4
# 7	1	3.8
# 8	7	26.9

**Table 4.** Preliminary Results of YSM WLDP Retrospective Evaluation Study

Variable	Min.	Max.	Mean (S.D.)
*As a result of completing YSM WLDP…*			
I have better understanding of my leadership vision	3	5	4.31 (0.72)
I have better appreciation of my leadership potential^a^	3	5	4.40 (0.62)^a^
I became more effective in my relationships with mentees & trainees	2	5	4.15 (0.72)
I became more effective relating to mentors and/or supervisors	2	5	4.12 (0.80)
I became more effective with my time management	2	5	3.58 (0.84)
I learned how to lead with my strengths	3	5	4.35 (0.55)
I strengthened my communication and interpersonal skills necessary for leadership in my field	3	5	4.31 (0.54)
My confidence in my ability to succeed in my field at Yale increased	2	5	4.12 (0.80)
My confidence in my capacity to be a leader in my field of academic medicine increased	2	5	4.27 (0.86)
My confidence in negotiating my needs at my workplace increased	2	5	4.04 (0.85)
My confidence in speaking up around gender issues increased	2	5	3.96 (0.94)
My confidence in navigating the leadership structure at YSM increased	2	5	3.84 (0.97)
My sense of engagement working at Yale increased	2	5	4.00 (0.98)
I believe I can make a greater impact in academic medicine now compared to before participating in YSM WLDP	2	5	4.23 (0.93)
*YSM WLDP Instructor & Course Materials*			
Instructor demonstrated expertise in subject	1	5	4.62 (0.88)
Instructor clearly explained concepts	3	5	4.76 (0.51)
Participant and instructor interaction was sufficient	3	5	4.72 (0.53)
Instructor was responsive to questions	4	5	4.92 (0.27)
The materials were clear and understandable	3	5	4.76 (0.51)
*Qualitative Items*			
What change(s) have you or do you plan to make in your leadership role(s) and/or capacity after attending this program?			
What other areas of leadership development might you like to learn about?			
What could we do to help you maintain your leadership skills?			
Please share any additional comments or feedback about the program.			

^a^ *n* = 25 for this question; one person answered “6” (not applicable), and her data were dropped so as to not inflate the mean.

Max., maximum; Min., minimum; S.D., standard deviation; YSM WLDP, the Yale School of Medicine Women’s Leadership Development Program.

## Results

Of the 62 program enrollees emailed about the evaluation, a total of 26 members (42%) of the WLDP cohorts participated in this evaluation. Most participants were 35–44 years old, heterosexual, White, non-Hispanic, U.S. citizens, full-time faculty, assistant professors, at Yale for almost five years, and had never received group or individual leadership development/career coaching prior to attending the WLDP. Further, participants in this study came from a range of academic departments and tended to draw from our later cohorts. For detailed information on participant demographics, please refer to Tables 2 and 3.

Overall, 14 (54%) of 26 participants were “extremely satisfied” with the YSM WLDP, while seven were “very satisfied,” and two each were “somewhat satisfied” and “somewhat dissatisfied” while one participant did not respond. Further, on a scale of 1 to 6 with *1 = Strongly Disagree*, *2 = Disagree*, *3 = Neither agree nor disagree*, *4 = Agree*, *5 = Strongly Agree*, and *6 = Not applicable*, most participants felt that as a result of completing the YSM WLDP, they had gained better appreciation of their leadership potential (*M* = 4.46, *S.D*. = 0.69); learned how to lead with their strengths (*M* = 4.35, *S.D*. = 0.55); acquired better understanding of their leadership vision (*M* = 4.31, *S.D*. = 0.72); strengthened the communication and interpersonal skills necessary for leadership in their field (*M* = 4.31, *S.D*. = 0.54); increased confidence in their capacity to be a leader in their field of academic medicine (*M* = 4.27, *S.D*. = 0.86); and believed they could subsequently make a greater impact in academic medicine (*M* = 4.23, *S.D*. = 0.93). Additionally, on a scale of 1 to 5 with *1 = Strongly Disagree* and *5 = Strongly Agree*, participants also indicated strong confidence in the instructor, noting that she was responsive to their questions (*M* = 4.92, *S.D*. = 0.51), explained the program concepts clearly (*M* = 4.76, *S.D*. = 0.51), and demonstrated expertise in the subject (*M* = 4.62, *S.D*. = 0.88). Considering the range of data (1 to 5) and normal standard deviation for all results indicating healthy variability, the means (all 3.5 or above) evidence robustness of the findings. For detailed information, please see Table 4.

Further, since starting or completing the YSM WLDP, all 26 participants reported using at least one of the leadership skills they learned in the program, and 21 participants recommended this course to others. Though 7 out of the 26 had completed the survey within weeks of completing this course, a total of 15 out of 26 participants still reported taking on new leadership roles. We hypothesize that more participants from the latest (eighth) cohort will likewise assume leadership roles in the future.

Participants provided their unique perspectives on the qualitative items. Most expressed an increased awareness of and confidence in leading with their strengths. As one participant wrote, “*I have gained confidence in leading from strength but also understand how to more effectively incorporate team members and look to highlight their complementary strengths.*” Another participant noted, “*I communicate more directly, advocate for what I need or when I need more time to perform tasks, I am more comfortable saying no or disagreeing and more confident about expressing my opinion*.” A third highlighted how the WLDP helped with “[establishing] *more boundaries, saying no, delegating tasks and negotiating for more protected time.”*

Participants provided recommendations to maintain their leadership skills, such as “*periodic reunions, refreshers, or reading/book recommendations*,” “*program alumni events to interact/network with other women leaders who have completed the program*,” and increased accountability through ongoing coaching, such as “*skills sessions to practice exercising skills; 6 month follow-up sessions with cohort for 2 years*.” Participants also recommended increasing the program length, making the program “*welcoming to gender diverse individuals*,” and “*consider offering an option for faculty of color to work with a consultant of color as being a faculty of color comes with unique challenges that a white consultant may not always know how to address.”*

Conclusively, participants appreciated attending the YSM WLDP and felt it had helped them learn critical leadership skills and enhance confidence in their leadership potential. This is reflected in participants’ quotes, “*I have attended other leadership programs, and this was by far the best program I have seen. This was time I looked forward to spending with peers. This always gave me a new skill, perspective or just even reinvigoration to be around some luminary young women in medicine*,” and “*The course was one of the best things I have ever done for my career.”*

## Discussion

In her critically acclaimed book *Playing Big*, leadership expert Tara Mohr summarizes the paradox women face when assuming positions of leadership:

Members of out-groups or low-status groups…are generally seen as only one or the other-competent *or* warm. This is the wider context for the double bind women face: It’s not just women but all stereotyped, low-power groups who are seen as either likable or effective, but not both.^48^ (p. 189)

The overarching purpose of the YSM WLDP is to help early-career women faculty navigate this bind by providing explicit instruction on building their leadership competencies and skills while also supporting their honest, authentic, and peer-supported navigation of the complexities of working within academic medicine. In building our program around the three pillars of anchoring oneself, cultivating skills and relationships, and embracing connection and generativity, we have aimed to foster faculty members’ individual development and collective support. The highlighting of coaching skills is another way in which we aimed to empower our faculty to help themselves and one another.

Outcomes from this preliminary study speak to the program’s impact, with participants reporting enhanced appreciation of their leadership potential, knowing how to lead with their strengths, and expanded leadership vision. Data (please see Table 4) also suggest acquisition of skills in pursuit of leadership aims, including participants’ strengthened communication and interpersonal skills, sense of confidence their capacity to be a leader in their field of academic medicine, and belief that they could make a greater impact in academic medicine after participating in the YSM WLDP. These findings are in line with other emerging studies about the effectiveness of leadership development programs for early-career women’s sense of career efficacy and acquisition of new skills.^38^

Qualitative responses also gave voice to the professional benefits accorded by the program. Participants noted concrete steps—drawing boundaries, leading with strengths, delegating, and advocating for themselves—that they now feel more equipped to put into place. Overall impressions of the program were equally positive, with participants noting the continual acquisition of new skills and appreciation of the collegiality of the group format. Such preliminary study findings have encouraged us to continue delivering this program to early-career women faculty, to consider how we may best provide for additional supports needed, and to enhance the rigor with which we will study the program’s impact.

The present study has several limitations. The number of participants (*n* = 26) is modest, and we have neither a control/comparison group nor a prepost, longitudinal design that would allow us to measure change over time. Due to the small number of participants from URiM backgrounds, we are also unable to parse the data by variables related to intersectionality (*i.e.,* participants’ race, ethnicity, sexual orientation, department of origin). This is of particular note given participants’ request that more attention be paid to the unique career challenges of women from traditionally underrepresented groups in medicine.

Our next steps strive to amend these various limitations. First, in the fall of 2024, we began *prospectively* measuring variables of interest including burnout, imposter syndrome, sense of belonging, and level of different leadership and communication skills. This longitudinal study design also includes data collection postprogram and at 3-, 6-and 12-month follow-up. Our hope is too more clearly distinguish the WLDP’s impact on early career women faculty’s career trajectory while more closely measuring changes to women’s perceived sense of collegiality, career efficacy, and personal leadership potential after completing the program.

Using administrative data from our Office of Institutional Research, we also plan to match outcomes for study participants regarding retention, promotion, and the assumption of leadership roles with those of early-career women faculty across our medical school. Once our sample size is large enough, we plan to explore the unique challenges and benefits that the program may offer to women of diverse backgrounds and lived experiences.

## Conclusion

Ultimately, we are interested in scaling our work and making it accessible to other institutions. If the promise of our initial outcomes holds upon further study, we would ideally like to make our program available outside of the Yale School of Medicine. Implementation would require the training of facilitators other than the first author, and also the infusion of resources by institutions that choose to adopt this program. Current per-participant cost for our program includes the $60 NovoEd license, approximately $90 for the self-report measures, and approximately $80 for books. This comes to about $230 per participant, or approximately $3700 annually, which our YSM Office of Diversity, Equity, & Inclusion currently covers. We understand that not all institutions can commit to deploying these resources consistently and that this is not a small sum to ask early-career faculty to cover directly either. Some ideas going forward include adapting the program so that it is delivered outside of the NovoEd platform and/or shifting the reading materials to exclusively those freely available in the public domain.

In the end, we believe that buy-in of leadership and the larger institution can make or break the fate of leadership development programming. We have been very fortunate that our dean and other key stakeholders have been willing to put resources behind this and other programming, and we hope the same will hold true at other centers of academic medicine. It is also our hope that papers like this one and the many reviewed herein will inspire women faculty to pursue their careers with courage, collegiality, and confidence, while also moving decisions-makers within academic medicine to support their women faculty through dynamic leadership development initiatives.

## Abbreviatios Used

ELAM: Executive Leadership in Academic Medicine
URiM: Underrepresented in Medicine
WLDP: Women's Leadership Development Program
YSM: Yale School of Medicine
